# Sensitivity of fever for diagnosis of clinical malaria in a Kenyan area of unstable, low malaria transmission

**DOI:** 10.1186/1475-2875-13-163

**Published:** 2014-04-30

**Authors:** Albino L Mutanda, Priscah Cheruiyot, James S Hodges, George Ayodo, Wilson Odero, Chandy C John

**Affiliations:** 1 School of Public Health and Community Development, Maseno University, Kisumu, Kenya; 2 School of Medicine, MasenoUniversity, Kisumu, Kenya; 3 Department of Pediatrics, University of Minnesota, Minneapolis, MN, USA; 4 Division of Biostatistics, School of Public Health, University of Minnesota, Minneapolis, USA; 5 Center for Global Health Research, Kenya Medical Research Institute, Kisumu, Kenya

**Keywords:** Malaria, *Plasmodium falciparum*, Symptom, Fever, Highland, Unstable transmission

## Abstract

**Background:**

Malaria in highland areas of Kenya affects children and adults. Local clinicians include symptoms other than fever when screening for malaria because they believe that fever alone does not capture all cases of malaria.

**Methods:**

Individuals who presented to dispensaries in a highland Kenya site of low, unstable malaria transmission from 2007–2011 with 1 or more of 11 symptoms were tested by microscopy for malaria. Clinical malaria was defined as asexual *Plasmodium falciparum* infection on peripheral blood smear in an individual with any screening symptom. Asymptomatic *P. falciparum* infection was assessed in a cohort at ten time points to determine the extent to which symptomatic episodes with parasitaemia might be attributable to baseline (asymptomatic) parasitaemia in the community.

**Results:**

3,420 individuals were screened for malaria, 634 < 5 years of age and 2,786 ≥ 5 years of age. For the diagnosis of clinical malaria, the symptom of fever had a sensitivity and specificity of 88.9% and15.4% in children <5 years, and 55.8% and 54.4% in children ≥5 years, respectively. Adding the symptom of headache increased sensitivity to 94. 4% in children <5 years and 96.8% in individuals ≥5 years, but decreased specificity to 9.9% and 11.6%, respectively, and increased the number of individuals who would be tested by 6% and 92%, respectively. No combination of symptoms improved upon the presence fever or headache for detection of clinical malaria. In the cohort of asymptomatic individuals, *P. falciparum* parasitaemia was infrequent (0.1%).

**Conclusion:**

In areas of low, unstable malaria transmission, fever is a sensitive indicator of clinical malaria in children <5 years, but not in older children and adults. Adding headache to fever as screening symptom increases sensitivity of detection in individuals ≥5 years old at the cost of decreased specificity. Screening for symptoms in addition to fever may be required to accurately capture all cases of clinical malaria in individuals ≥5 years old in areas of low malaria transmission.

## Background

Fever is the universal screening symptom for malaria in research studies [1-4], but the highland areas of Kenya are unusual in that they have historically had little malaria incidence. Malaria appears to have been introduced by rail and travel of persons from endemic populations to highland areas for tea planting and harvesting in the early 1900s [5,6]. Thus, families in this area have not had generations of exposure to malaria, as have families in nearby areas, such as Nyanza Province [7,8]. This is supported by the relative lack of sickle cell trait and G6PD deficiency in individuals in the highland areas as compared to individuals in lowland areas [9]. Thus symptoms and immunity in this population may differ from those in populations with a long history of exposure (e.g. Nyanza Province) and in populations with no exposure for decades (e.g. the United States and Europe). In the highland sites of Kipsamoite and Kapsisiywa, in Nandi North District, Rift Valley Province, Kenya, where our research team has conducted malaria research for the past decade, local clinical officers state that they must inquire about symptoms in addition to fever to capture all cases of malaria.

Kipsamoite and Kapsisiywa experienced an atypical situation in 2007, when widespread indoor residual spraying (IRS) with long-lasting insecticides and introduction of artemisinin combination therapy with artemether-lumefantrine (Coartem©) led to a 14-month period with no clinical cases of malaria and possible interruption of malaria transmission [10]. After this period, asymptomatic parasitaemia has been infrequent in these communities, providing a unique opportunity to study the sensitivity and specificity of fever and other symptoms for *P. falciparum* parasitaemia, which at this point occur almost exclusively in individuals with symptoms that lead them to seek evaluation and treatment at the health dispensaries on site. Individuals with any of eleven symptoms considered consistent with malaria by study clinical officers were tested for malaria by microscopy. In the present study, sensitivity and specificity of individual symptoms and combinations of symptoms for clinical malaria were assessed, with the primary definition of clinical being *P. falciparum* parasitaemia in any symptomatic individual. Sensitivity and specificity of individual symptoms and combinations of symptoms for alternate definitions of clinical malaria were also assessed, including: (1) *P. falciparum* parasitaemia with measured fever (axillary temperature ≥37.5°C), (2) *P. falciparum* parasitaemia ≥ 1,000 parasites/μL with measured fever, or (3) *P. falciparum* parasitaemia ≥ 4,000 parasites/μL with measured fever.

## Methods

### Study population and recruitment

The study was conducted in the Kenyan highland sites of Kipsamoite (population ~4,800) and Kapsisiywa (population ~4,000), Nandi County, from 2007 to 2011. Elevation ranges from 1,887 m to 2,132 m at these sites. The area has seasonal, unstable malaria transmission and a history of past malaria epidemics and a recent possible interruption of malaria transmission from April 2007 to May 2008, with no clinical cases of malaria identified in that period [10]. Persons living in these areas are predominantly Nandi, a Kalenjinsub-tribe. Written informed consent for study participation was obtained from heads of households in the area for demographic studies and from individuals (or parents or guardians of persons <15 years of age) for other studies. Ethical approval for the study was obtained from the Kenya Medical Research Institute National Ethical Review Committee and the Institutional Review Board for Human Studies at the University of Minnesota.

### Demography and surveillance for clinical malaria and asymptomatic parasitaemia

Demographic surveys and malaria surveillance were performed in these areas. Demography of households was done annually. Surveillance for clinical malaria was conducted at the Kenyan Ministry of Health dispensaries in both sites, which are the only health care facilities within the area. Free malaria diagnosis and treatment were available to all persons within the area. Individuals with symptoms consistent with malaria who did not have a clear alternative diagnosis were tested for *Plasmodium* species by microscopic examination of blood smears. The symptoms were determined by local clinical officers and included fever, chills, headache, loss of appetite, vomiting, jaundice, diarrhea, backache, joint pains, nausea, and severe malaise. Microscopy was performed as previously described [11] with2 independent readings and a third reading for slides with discordant results. All persons with positive blood smears for *P. falciparum* or *Plasmodium malariae*, the only *Plasmodium* species documented in this area, were treated with artemether-lumefantrine (Coartem©). In order to determine the extent to which baseline asymptomatic parasitaemia in the community might contribute to parasitaemia detected in symptomatic individuals, ten surveys for asymptomatic *P. falciparum* parasitaemia were conducted in a cohort of randomly selected individuals from the sites between2007 and 2011.

### Statistical analysis

Since clinical immunity to malaria differs in most populations in children <5 years of age as compared to individuals ≥5 years of age, analyses were done separately for individuals in these two age groups. Frequency of symptoms in individuals with versus without parasitaemia was compared by c^2^ testing. Logistic regression was conducted to compare odds of having *P. falciparum* parasitaemia or *P. falciparum* parasitaemia with a measured temperature ≥37.5°C with a specific symptom. Data were analysed using Stata Version 11 (Stata Corp, College Station, Texas, USA) and R(R Foundation, Wien, Austria).

## Results

### Sensitivity and specificity of specific symptoms for clinical malaria

During the study period, only 3/3,420 smears done were positive for *P. malariae* (0.09%)*,*so only the results for individuals positive for *P. falciparum* were analysed*.* From 2007 to 2011, 3,420 individuals were screened for malaria, and *P. falciparum* parasitaemia was detected in 224 individuals (6.5%), including 36/634 children <5 years (5.7%) and 188/786 individuals ≥5 years (6.7%, P = 0.33). The median parasite density was 30,700 (IQR 10,770 - 135390), and did not differ in children <5 years (768 [IQR 295–3202]) compared to individuals ≥5 years (19,420 [IQR 3230–56,920], *P* = 0.32).

The frequency of individual symptoms for clinical malaria, defined as *P. falciparum* parasitaemia in a person with any of the eleven symptoms, along with the sensitivity and specificity of each symptom, are presented in Table 1 for children <5 years and in Table 2 for individuals ≥5 years. Findings for clinical malaria defined as symptomatic *P. falciparum* parasitaemia with measured fever (axillary temperature ≥ 37.5°C) are summarized in Additional files 1 and 2. Negative and positive predictive values are not shown, because the low frequency of *P. falciparum* parasitaemia in participants seen at these clinics led to high negative predictive values (all >96%) and low positive predictive values (all <16%) for all symptoms assessed.

**Table 1:** **Frequency, sensitivity and specificity of symptoms for clinical malaria (symptomatic *Plasmodium falciparum *** **parasitaemia) in children <5 years of age**

**Symptoms**	**Pf pos,**^**a**^**n (%)**	**Pf neg,**^**b**^**n (%)**	***P value***	**Sensitivity (%)**	**Specificity (%)**
Fever	32 (89)	506 (85)	0. 49	88. 9	15. 4
Appetite loss	25 (64)	323 (54)	0. 07	69. 4	46
Headache	19 (53)	191 (32)	0. 01	52. 8	68. 1
Vomiting	11 (31)	237 (40)	0. 28	30. 6	60. 4
Chills	10 (28)	56 (9)	<0. 001	27. 8	90. 6
Malaise	7 (19)	53 (9)	0. 04	19. 4	91. 1
Diarrhea	5 (14)	121 (20)	0. 35	13. 9	79. 8
Nausea	3 (8)	30 (5)	0. 38	8. 3	95
Joint pains	1 (3)	12 (2)	0. 75	2. 8	98
Jaundice	0 (0)	7 (1)	0. 51	0	98. 8
Backache	0 (0)	8 (1)	0. 49	0	98. 7

*Abbreviations*: *Pf P. falciparum*, *pos* positive, *neg* negative.

^a^Total N for Pf pos, N = 36.

^b^Total N for Pf neg, N = 598.

**Table 2:** **Frequency, sensitivity and specificity of symptoms for clinical malaria (symptomatic *Plasmodium falciparum *** **parasitaemia) in individuals ≥ 5 years of age**

**Symptoms**	**Pf pos,**^**a**^**n (%)**	**Pf neg,**^**b**^**n (%)**	***P value***	**Sensitivity (%)**	**Specificity (%)**
Headache	167 (89)	2055 (79)	0. 001	88. 8	20. 9
Fever	105 (56)	1185 (46)	0. 01	55. 9	54. 4
Appetite loss	69 (37)	834 (32)	0. 19	36. 7	67. 9
Chills	68 (36)	457 (18)	<0. 001	36. 2	82. 4
Joint pains	57 (30)	641 (25)	0. 08	30. 3	75. 3
Vomiting	48 (26)	460 (18)	0. 01	25. 5	82. 3
Malaise	36 (19)	325 (13)	0. 01	19. 1	87. 5
Backache	35 (19)	494 (19)	0. 89	18. 6	81
Nausea	26 (14)	447 (17)	0. 23	13. 8	82. 8
Diarrhoea	6 (3)	323 (12)	<0. 001	3. 2	87. 6
Jaundice	2 (1)	25 (1)	0. 89	1. 1	99

^a^Total N for Pf pos, N = 188.

^b^Total N for Pf neg, N = 2598.

Symptoms with the greatest specificity lacked sensitivity (e.g. chills in children < 5 years had a specificity 91%, but a sensitivity of only 28%), and those with the greatest sensitivity lacked specificity (e.g. fever in children <5 years had a sensitivity of 89%, but a specificity of only 15%). Of particular interest, fever in individuals ≥ 5 years was neither sensitive (56%) nor specific (54%).

### Odds of clinical malaria with specific symptoms

Table 3 summarizes the odds ratios for presence of each symptom for clinical malaria in children <5 years and individuals ≥ 5 years. Individuals <5 years and ≥5 years with *P. falciparum* parasitaemia were significantly more likely to have headache, malaise, and chills than those without parasitaemia. Individuals ≥5 years with *P. falciparum* parasitaemia were also significantly more likely to have fever and vomiting, and significantly less likely to have diarrhoea, than those without parasitaemia. Findings for the outcome of *P. falciparum* parasitaemia with measured fever (axillary temperature ≥37.5°C) were very similar and are summarized in Additional file 3.

**Table 3:** **Odds ratios of symptoms for prediction of clinical malaria (symptomatic *Plasmodium falciparum *** **parasitaemia)**

**Predictor**	**<5 years**			**≥5 years**		
	**OR**	**95% CI**	**P**	**OR**	**95% CI**	**P**
Fever	1. 96	0. 59–6. 53	0. 27	1. 47	1. 09–1. 97	0. 01
Headache	2. 54	1. 28–5. 04	0. 01	2. 04	1. 3–3. 21	0. 002
Appetite loss	2. 14	1. 01–4. 53	0. 05	1. 21	0. 89–1. 65	0. 22
Vomiting	0. 7	0. 34–1. 46	0. 34	1. 74	1. 25–2. 43	0. 001
Chills	3. 88	1. 77–8. 49	0. 001	2. 63	1. 93–3. 51	<0. 001
Jaundice	1	-	-	1. 08	0. 25–4. 60	0. 92
Diarrhoea	0. 5	0. 18–1. 46	0. 21	0. 23	0. 1–0. 52	<0. 001
Backache	1	-	-	0. 95	0. 65–1. 38	0. 78
Joint pains	1. 44	0. 12–11. 39	0. 73	1. 29	0. 93–1. 78	0. 13
Nausea	1. 78	0. 52–6. 14	0. 36	0. 79	0. 52–1. 2	0. 27
Malaise	2. 58	1. 07–6. 18	0. 03	1. 67	1. 15–2. 44	0. 01

### Sensitivity and specificity of combinations of symptoms for P. falciparum parasitaemia

To assess whether any combination of symptoms could improve on fever for sensitivity and specificity of detection of clinical malaria, a three-step process was undertaken: (1) candidate subsets of symptoms to consider were selected; (2) for each candidate subset of symptoms, a logistic regression model was fitted including those symptoms; (3) the logistic regression results were used to define rules and estimate their sensitivity and specificity. The results of this assessment are summarized in Table 4 (for individuals <5 years) and Table 5 (for individuals ≥ 5 years).

**Table 4:** Sensitivity and specificity of combinations of symptoms for different clinical malaria definitions in children <5 years of age

A. Symptomatic *P. falciparum* parasitaemia			
Predictors	Rule	Sensitivity (%)	Specificity (%)
Fever	Fever	88. 9	15. 4
F, C, H, M, A	Any of F, C, H, M, A	97. 2	5. 5
F, C, H, M	Any of F, C, H, M	94. 4	8. 0
F, C, H	Any of F, C, H	94. 4	8. 9
F, C	F or C	88. 9	13. 4
F, H	F or H	94. 4	9. 9
B. Symptomatic *P. falciparum* parasitaemia plus T ≥ 37.5°C			
Predictors	Rule	Sensitivity	Specificity
Fever	Fever	92. 0	11. 7
F, C, H, M, A	Any of F, C, H, M, A	100. 0	5. 6
C, H, M, A	Any of C, H, M, A	96. 0	25. 6
C, H, M	Any of C, H, M	80. 0	55. 8
F, H	F or H	100. 0	6. 5
C. Symptomatic *P. falciparum* parasitaemia ≥1,000parasites/μL plus T ≥ 37.5°C			
Predictors	Rule	Sensitivity	Specificity
F	Fever	100. 0	19. 3
F, H	F or H	100. 0	13. 3
D. Symptomatic *P. falciparum* parasitaemia ≥4,000 parasites/μL plus T ≥ 37.5°C			
Predictors	Rule	Sensitivity	Specificity
F	Fever	100. 0	19. 5
F, H	F or H	100. 0	13. 3

*Abbreviations*: *F* fever, *C* chills, *H* headache, *M* Malaise, *A* appetite.

**Table 5:** Sensitivity and specificity of combinations of symptoms for different clinical malaria definitions in individuals ≥5 years of age

A. Symptomatic *P. falciparum* parasitaemia			
Predictors	Rule	Sensitivity	Specificity
F	Fever	55. 2	54. 4
C, F, M, H, V, ~D	Any of F, C, ~D, V, H	100. 0	2. 4
C, F, M, ~D	Any of F, C, ~D	98. 5	5. 6
F, ~D	Either of F, ~D	98. 5	6. 6
~D	~D	96. 9	12. 5
C, F, M, ~D	≥ 2 of C, F, M, ~D	73. 7	43. 5
F, H	F or H	96. 8	11. 5
B. Symptomatic *P. falciparum* parasitaemia plus T ≥ 37.5°C			
Predictors	Rule	Sensitivity	Specificity
F	Fever	64. 7	54. 5
C, F, M, H, V, ~D	Any of C, F, M, H, V, ~D	100. 0	2. 1
C, F, M, ~D	Any of C, F, M, ~D	98. 3	4. 9
F, ~D	F,~D	98. 3	6. 5
~D	~D	96. 6	12. 2
C, F, M, ~D	≥ 2 of C, F, M, ~D	82. 8	40. 9
F, H	F or H	96. 6	7. 6
C. Symptomatic *P. falciparum* parasitaemia ≥1,000 parasites/μL plus T ≥ 37.5°C			
Predictors	Rule	Sensitivity	Specificity
F	Fever	69. 6	58. 4
F, H	F or H	100. 0	23. 0
D. Symptomatic *P. falciparum* parasitaemia ≥4,000 parasites/μL plus T ≥ 37.5°C			
Predictors	Rule	Sensitivity	Specificity
F	Fever	64. 8	54. 2
C, F, M, V, ~D, H	Any of C, F, M, V, ~D, H	100. 0	2. 0
C, F, M, V, ~D	Any of C, F, M, V, ~D	97. 7	4. 8
~D	~D	95. 5	12. 1
C, F, M, V	Any of C, F, M, V	84. 1	34. 8
F, H	F or H	100. 0	22. 9

*Abbreviations*: *F* fever, C *chills*, *H* headache, *M* Malaise, *V* vomiting, *~D* No diarrhoea.

As documented in Tables 4 and 5, no combination of symptoms had higher sensitivity than presence of fever or headache for detection of clinical malaria, although this combination had poor specificity. Some symptom groupings had improved specificity, but always at the cost of sensitivity. A combination of fever or headache achieved a sensitivity of 94% and 97% for individuals <5 years and ≥5 years respectively (Tables 4 and 5), but at the cost of greatly decreased specificity (6. 5%) in individuals ≥5 years. The addition of headache increased the number of children <5 years who would be tested for *P. falciparum* infection by only two persons (6%), but increased the number of individuals ≥5 years who would be tested by 1,190 persons (92%).

To assess whether more stringent criteria for a definition of clinical malaria would result in different findings, sensitivity and specificity of symptom combinations was assessed for a number of stricter definitions of clinical malaria, as noted in Tables 4 and 5. Similar patterns were seen for all alternate definitions of clinical malaria. Finally, measured fever was incorporated in the clinic into the “symptom” predictor variable. For the primary definition of clinical malaria (symptomatic *P. falciparum* parasitaemia), having a history of fever *or* measured fever in the clinic had a sensitivity and specificity of 94% and 11%, respectively, for children <5 years, and 78% and 44%, respectively, for persons ≥5 years.

### Prevalence of asymptomatic parasitaemia

Six hundred and four randomly selected individuals from the site were assessed for presence of asymptomatic parasitaemia in July and November 2007, March, August and October 2008, February, April, August and October 2009, and May 2010. Due to movement of individuals during the study period, different numbers were tested at each time point, but during the three-year period, only five persons out 5,195 tested had asymptomatic parasitaemia (0.1%), with a median parasite density of 240 (interquartile range [IQR], 200–2,560).

## Discussion

Fever is the universal screening tool for clinical malaria in research studies. This is likely appropriate in children <5 years, who constitute the majority of malaria cases in highly endemic areas, because they are more likely to develop fever when they have infection than are older individuals. However in areas such as the highland area of the present study, a substantial proportion of clinical malaria occurs in individuals ≥5 years [12] and local clinicians state that fever alone does not detect all cases of malaria in these individuals. The present study supports this claim. Almost half of individuals ≥ 5 years who came to the clinic with symptoms of illness and who had *P. falciparum* parasitaemia would not have been captured as malaria cases had history of fever been the sole screening criterion. A recent study by D’Acremont *et al.* found that in one area of Tanzania, malaria was a less common cause of fever than other infections, notably respiratory viral infection [13]. The present study answers the inverse question (does fever as a symptom capture all cases of clinical malaria?), and finds that in areas of low malaria transmission, fever alone may not be an adequate screening criterion for malaria.

A history of fever may be absent in individuals ≥5 years with parasitaemia for several reasons. Fever may be less evident in older children and adults than in younger children, whose temperature may be checked or noted by their parent. This is supported by the only modest increase in sensitivity of a history of fever among individuals ≥5 years who had a measured fever at the clinic. In areas of higher transmission, parasitaemia could be incidental rather than causative of symptoms. However in this area, based on the surveys done, one would expect <0.2% of individuals to have asymptomatic parasitaemia, so incidental parasitaemia should account for only a tiny fraction of the parasitaemia in symptomatic individuals seen in the clinic. In addition, history of fever had sensitivity only 65% even for clinical malaria defined as parasitaemia > 4,000 parasites/μL and a measured fever at the clinic, individuals who in this context indisputably have clinical malaria. The Nandi words for each symptom, including fever, were carefully reviewed with our study field assistants, and verified by the clinical officers and the local advisory group, and the symptom of fever was highly sensitive for detection of parasitaemia in young children, so confusion about the term is unlikely. The most likely explanation seems to be that other symptoms precede fever in older individuals. These symptoms bring them to the dispensary, at which time some have measured fever but others remain afebrile. Given the clinical course of malaria, all of these individuals were likely to have a fever eventually, but in a dispensary setting, particularly in an area of low transmission that is working toward elimination, one wants to detect malaria early and accurately. Individuals with malaria who have symptoms other than fever, but are not tested or treated because fever is the sole screening criterion, are likely to go outside the health system, and potentially receive inappropriate treatment.

Unfortunately, no symptom combination emerged that would allow us to detect >90% cases of parasitaemia in individuals ≥5 years without screening many more uninfected individuals. A previous study in highland Uganda found that a history of fever or presence of measured fever at the clinic had a sensitivity of 95.7% and a specificity of 25.5% [14], but the findings were not separated by age. In the present study, reported or measured fever had a sensitivity and specificity of only 78% and 44%, respectively, for individuals ≥5 years, so reported or measured fever alone would not be adequate for screening in older individuals. The simplest combination of symptoms that achieved approximately 95% sensitivity in individuals ≥ 5 years was fever or headache (sensitivity 96.8%), but screening with these two symptoms increased the number of individuals ≥ 5 years to be screened to almost twice what was required with fever (from 1,290 to 2,480). Headache has been noted in the travel medicine literature to be a frequent complaint in adult travellers with malaria, though most travellers with malaria also have fever [15]. The presence of fever early in travellers, but not in this highland population, may be due to partial immunity from prior exposure in this population, resulting parasitaemia that does not produce immediate fever but does produce other lower-grade symptoms such as headache.

Definitions that relied on more symptoms were more complex, but did not have better sensitivity than asking about fever or headache, so in the present study site, fever or headache will be used as screening symptoms because they are the best single set of screening symptoms that will capture ≥95% individuals with clinical malaria. An alternative would be to wait for individuals to come in for a repeat visit, since malaria likely will eventually result in fever in all individuals, but this would lead to missed chances to diagnose malaria, to prolonged parasitaemia in symptomatic individuals, and potentially to lost confidence in the health dispensary. The findings of the present study may have applicability to other sites of unstable transmission, and in these areas analysis of symptoms should be done to determine if screening for fever alone can detect >95% of individuals who have clinical malaria.

An interesting incidental finding is that the *absence* of diarrhoea had 96% sensitivity for detection of clinical malaria in individuals ≥5 years. The finding probably reflects a combination of the low incidence of clinical malaria at this site, the frequency of diarrhoea as an illness, the multiple other causes of diarrhoea in this area, and the relatively low frequency of diarrhoea as a symptom of malaria. The study findings contrast with those of Coldren *et al*., who found a modest association between *P. falciparum* parasitaemia and a history of diarrhoea in adults from three areas of Kenya with differing malaria transmission intensity [16]. The differences may in part reflect different populations: the present study was restricted to an area of very low endemicity and included children as well as adults.

## Conclusions

The present study documents that in a highland area of unstable transmission, where asymptomatic parasitaemia is rare, the symptom of fever alone is adequate for detection of clinical malaria in individuals <5 years but not in individuals ≥5 years. In areas of high malaria transmission, malaria occurs most often in children and is deadliest in children [17-21], so a history of fever, which detects almost all cases of malaria in young children, is a reasonable screening criterion. However, as more areas become areas of low transmission, disease in adults and adolescents may form a larger part of the clinical burden, and additional symptoms may be needed to identify all potential cases. Individuals in areas of unstable, low transmission differ from individuals in areas of high transmission, who have strong clinical immunity, and travellers from non-malaria endemic areas, who have little or no immunity. The intermediate initial presentation of low-grade symptoms, such as headache, may reflect their partial clinical immunity. Studies in other areas of unstable transmission should be conducted to confirm the results of the present study.

## Competing interests

The authors declare that they have no competing interests.

## Authors’ contributions

ALM conceptualized the study, conducted literature review, performed data analysis and draftedthe manuscript. PC did data cleaning and initial data analysis. GA and WO reviewed the analysis and manuscript and provided key comments on manuscript revision. JSH conducted data analysis and helped to draft the initial and final manuscript. CCJ provided the study design, and oversaw data analysis, programme implementation, drafting of manuscript, and review of the manuscript. All authors read and approved the final manuscript.

## Supplementary Material

Additional file 1 **Frequency, sensitivity and specificity of particular symptoms for symptomatic *Plasmodium falciparum *** **parasitaemia with plus a measured axillary temperature ≥37.5°C in children <5 years of age.** Click here for file

Additional file 2 **Frequency, sensitivity and specificity of particular symptoms for symptomatic *Plasmodium falciparum *** **parasitaemia plus a measured axillary temperature ≥37.5°C in individuals ≥5 years of age.** Click here for file

Additional file 3 **Odds ratios of particular symptoms for prediction of symptomatic *P. falciparum *** **parasitaemia with a measured axillary temperature ≥37.5°C.** Click here for file
