# Supplementary material for: Sensitivity of fever for diagnosis of clinical malaria in a Kenyan area of unstable, low malaria transmission
Source: Malar J. 2014 Apr 30;13:163. doi: 10.1186/1475-2875-13-163 (PMC4021053; doi:10.1186/1475-2875-13-163)
Supplement: Additional file 1 — Frequency, sensitivity and specificity of particular symptoms for symptomatic Plasmodium falciparum parasitaemia with plus a measured axillary temperature ≥37.5°C in children <5 years of age. [file 1475-2875-13-163-S1.doc]

Additional file 1. Frequency, sensitivity and specificity of particular symptoms for symptomatic *Plasmodium falciparum* parasitemia with plus a measured axillary temperature ≥37.5°C in children <5 years of age.

| Symptoms | Pf. pos,^a^  n (%) | Pf. neg,^b^  n (%) | *P value* | Sensitivity  (%) | Specificity  (%) |
| --- | --- | --- | --- | --- | --- |
| Fever | 29 (94) | 228 (90) | 0.5 | 93.5 | 10.2 |
| Appetite loss | 22 (71) | 134 (53) | 0.05 | 71.0 | 47.2 |
| Headache | 17 (55) | 83 (33) | 0.02 | 54.8 | 67.3 |
| Vomiting | 6 (19) | 48 (19) | 0.95 | 32.3 | 61.8 |
| Chills | 10 (32) | 31 (12) | 0.003 | 32.3 | 87.8 |
| Malaise | 7 (23) | 29 (11) | 0.08 | 22.6 | 88.6 |
| Diarrhea* | 4 (13) | 39 (16) | 0.72 | 12.9 | 84.6 |
| Nausea | 2 (6) | 14 (6) | 0.83 | 6.5 | 94.5 |
| Joint pains | 1 (3) | 6 (2) | 0.77 | 3.2 | 97.6 |
| Jaundice | 0 (0) | 3 (1) | 0.54 | 0 | 98.8 |
| Backache | 0 (0) | 3 (1) | 0.54 | 0 | 98.8 |

Abbreviations: Pf., P. falciparum; pos, positive; neg, negative;

a. Total N for Pf pos, N= 31,

b. Total N for Pf neg, N= 254,
