# Supplementary material for: Sensitivity of fever for diagnosis of clinical malaria in a Kenyan area of unstable, low malaria transmission
Source: Malar J. 2014 Apr 30;13:163. doi: 10.1186/1475-2875-13-163 (PMC4021053; doi:10.1186/1475-2875-13-163)
Supplement: Additional file 2 — Frequency, sensitivity and specificity of particular symptoms for symptomatic Plasmodium falciparum parasitaemia plus a measured axillary temperature ≥37.5°C in individuals ≥5 years of age. [file 1475-2875-13-163-S2.doc]

Additional file 2. Frequency, sensitivity and specificity of particular symptoms for symptomatic *Plasmodium falciparum* parasitemia plus a measured axillary temperature ≥37.5°C in individuals≥5 years of age.

| Symptoms | Pf. pos,^a^  n (%) | Pf. neg,^b^ n (%) | *P value* | Sensitivity % | Specificity % |
| --- | --- | --- | --- | --- | --- |
| Headache | 120 (89) | 439 (82) | 0.04 | 89.1 | 18.4 |
| Fever | 83 (61) | 326 (61) | 0.85 | 61.3 | 39.5 |
| Appetite loss | 51 (38) | 196 (36) | 0.77 | 38 | 63.7 |
| Chills | 49 (36) | 125 (23) | 0.002 | 35.8 | 76.7 |
| Joint pains | 42 (31) | 135 (25) | 0.16 | 30.7 | 74.7 |
| Vomiting | 34 (25) | 114 (21) | 0.32 | 26.3 | 81.2 |
| Malaise | 28 (21) | 74 (14) | 0.04 | 20.4 | 86.2 |
| Backache | 24 (18) | 71 (13) | 0.17 | 17.5 | 86.8 |
| Nausea | 18(13) | 67 (12) | 0.78 | 13.9 | 87.7 |
| Diarrhea* | 6 (4) | 86 (16) | <0.001 | 4.4 | 84 |
| Jaundice | 2 (1) | 5 (1) | 0.57 | 1.5 | 99.1 |

Abbreviations: Pf., P. falciparum; pos, positive; neg, negative;

a. Total N for Pf pos, N= 135

b. Total N for Pf neg, N= 538
