# Supplementary material for: Sensitivity of fever for diagnosis of clinical malaria in a Kenyan area of unstable, low malaria transmission
Source: Malar J. 2014 Apr 30;13:163. doi: 10.1186/1475-2875-13-163 (PMC4021053; doi:10.1186/1475-2875-13-163)
Supplement: Additional file 3 — Odds ratios of particular symptoms for prediction of symptomatic P. falciparum parasitaemia with a measured axillary temperature ≥37.5°C. [file 1475-2875-13-163-S3.doc]

Additional file 3.Odds ratios of particular symptoms for prediction of symptomatic *P. falciparum* parasitemia with a measured axillary temperature ≥37.5°C.

| **Predictor** | **Age** | | | | | |
| --- | --- | --- | --- | --- | --- | --- |
|  | **<5 years** | | | **≥5 years** | | |
|  | **OR** | **95% CI** | **P** | **OR** | **95% CI** | **P** |
| Fever | 1.65 | 0.37-7.33 | 0.51 | 1.04 | 0.70-1.53 | 0.85 |
| Headache | 2.50 | 1.18-5.32 | 0.02 | 1.8 | 1.01-3.22 | 0.05 |
| Appetite loss | 2.19 | 0.97-4.94 | 0.06 | 1.06 | 0.72-1.56 | 0.77 |
| Vomiting | 1.03 | 0.40-2.65 | 0.95 | 1.25 | 0.81-1.94 | 0.32 |
| Chills | 3.43 | 1.48-7.95 | 0.004 | 1.88 | 1.26-2.82 | 0.002 |
| Jaundice | 1 | - | - | 1.6 | 0.31-8.35 | 0.58 |
| Diarrhea | 0.82 | 0.27-2.46 | 0.72 | 0.24 | 0.10-0.57 | 0.001 |
| Backache | 1 | - | - | 1.42 | 0.86-2.36 | 0.17 |
| Joint pains | 1.38 | 0.16-11.84 | 0.77 | 1.35 | 0.89-2.04 | 0.16 |
| Nausea | 1.18 | 0.26-5.46 | 0.83 | 1.08 | 0.62-1.89 | 0.73 |
| Malaise | 2.26 | 0.90-5.72 | 0.08 | 1.64 | 1.01-2.66 | 0.04 |
